# Effect of awareness training to frontline health workers and the use of e-based technology on reporting of brucellosis cases in selected pastoral communities, Tanzania: a quasi-experimental study

**DOI:** 10.1186/s42522-023-00084-3

**Published:** 2023-10-11

**Authors:** Belinda Joseph Mligo, Calvin Sindato, Richard B Yapi, Mpoki Mwabukusi, Coletha Mathew, Ernatus M Mkupasi, Esron D Karimuribo, Rudovick R Kazwala

**Affiliations:** 1 College of Veterinary Medicine and Biomedical Sciences, Sokoine University of Agriculture, P.O. Box 3015, Morogoro, Tanzania; 2 SACIDS Foundation for One Health, Sokoine University of Agriculture, P.O. Box 3297, Morogoro, Tanzania; 3 National Institute for Medical Research, Tabora Research Centre, Tabora, Tanzania; 4 Centre Suisse de Recherches Scientifiques en Côte d’Ivoire, Abidjan, Côte d’Ivoire; 5 Centre d’Entomologie Médicale et Vétérinaire, Université Alassane Ouattara, Bouaké, Côte d’Ivoire

**Keywords:** Knowledge, Practice, AfyaData, Reporting, Frontline health workers, One health

## Abstract

**Introduction:**

Brucellosis is a serious community health problem and endemic disease in Tanzania in both humans and animals. Frontline health workers (FHWs) play a vital role in reporting and hence prevent and control brucellosis in rural settings. This study aims to evaluate the effect of awareness training to frontline health workers and use of electronic technology (e- technology) on reporting of brucellosis cases.

**Methods:**

A quasi-experimental design was implemented in two pastoral communities in eastern part of Tanzania with one as control and another as treatment involving 64 FHWs who were purposively selected from May 2020 to December 2020. A total of 32 FHWs from treatment pastoral community were purposively selected for awareness training, rapid diagnosis using Rose Bengal test (RBT) and use of electronic technology (AfyaData app) for brucellosis reporting while nothing was done in control community. Before and after training information about their knowledge, attitude and practices were collected from all participants using a structured questionnaires uploaded in the mobile phone powered by AfyaData application. Blood samples were collected from 141 febrile patients attending the selected facilities in treatment community. Serum obtained from collected blood were analyzed using RBT and Competitive Enzyme Linked Immunosorbent Assay (c-ELISA) for brucellosis screening and confirmatory, respectively. Results from this analysis were reported back to the health facility using AfyaData app. Chi-square was used to analyze categorical variables and t-test and/Anova test was used to assess the effectiveness of the intervention.

**Results:**

Results revealed that before the training majority of the participants were ignorant about brucellosis, although they had good attitude towards brucellosis prevention. Participant’s awareness, practice and attitude increased significantly (p = 0.003, p = 0.001, p = 0.032) respectively, after the intervention. Total of 17(12.1%) patients were positive on RBT and four (2.8%) were confirmed by c-ELISA. AfyaData app was proven to provide quick reports regarding brucellosis in the study area.

**Conclusion:**

The training program was effective in increasing the level of knowledge and practice about brucellosis. Electronic based technology (AfyaData app) improved the reporting of brucellosis cases. There is a need for the use of electronic based technology to improve timely management of brucellosis in pastoral communities. Also, continuous training on FHWs regarding the disease is needed to improved their awareness and practices.

**Supplementary Information:**

The online version contains supplementary material available at 10.1186/s42522-023-00084-3.

## Introduction

Brucellosis is a zoonotic disease which causes severe illness in human and substantial economic losses in livestock production [1, 2]. The main causative agent is *Brucella* and in humans the disease is mostly caused by *B. abortus, B. melitensis, B. suis*, [3]. Humans acquire the infection through ingestion of contaminated animal products, inhalation of contaminated airborne particulates, or direct contact with infected animals or their products [1]. Human brucellosis is characterized by acute febrile illnesses that can progress to a chronic disease characterized by flu-like symptoms and musculoskeletal pain [4]. Also, development of focal (e.g., joint, pulmonary, gastrointestinal, hepatobiliary, genitourinary and neurological) complications is common and influenced by the length of time before diagnosis and initiation of treatment [5]. Treatment of human brucellosis requires long courses of combined antibiotics and whilst rates of relapse are low with the best regimes [6] compliance in resource-limited areas is often difficult to achieve.

Healthcare facilities and laboratories in low-resource settings where brucellosis is endemic face several challenges in the diagnosis of human brucellosis. The suggested diagnostic methods (e.g., culture and serological testing of paired sera using the SAT are technically demanding, have relatively slow improvement times, are expensive, and are often not available in many endemic settings [7–9]. Its diagnosis is further complicated by the fact that it shares symptoms with malaria and typhoid fever which are common cause of fever in sub-Saharan Africa [10]. The clinical presentation of brucellosis in humans is variable and unspecific, and thus, laboratory corroboration of the diagnosis is essential for the patient’s proper treatment [11]. Brucellosis might be poorly diagnosed due to poor health facilities, diagnostic facilities and limited awareness of the disease among medical practitioner [12]. Knowledge and awareness about brucellosis among health workers is considered to be an important aspect for the control of the disease, both in humans and animals [13]. Healthcare provider awareness on brucellosis in Tanzania is reported to be low [14].

According to WHO, there is high possibility for mobile technologies to enhance healthcare and public health service delivery, most specific in resource poor settings [15]. Successful surveillance depends on timely and full gathering of information to assess disease status, determine appropriate control strategies, and monitor their impact [15]. In Tanzania, the guidelines for surveillance and reporting of prioritized diseases, recommend the use of the electronic, Integrated Disease Surveillance and Reporting system (IDSR) [16]. The IDSR guidelines were first incorporated into the Tanzanian health system in 2001 and included 13 priority diseases. In 2011, the national IDSR guidelines were revised to include surveillance of 34 priority diseases and conditions in its second phase [17]. Although brucellosis is not a notifiable disease, it is a priority zoonosis in Tanzania, that should be reported at health facilities [16]. Nevertheless, despite these goals, the IDSR system is not fully integrated into health facility information management systems, especially in rural, primary health facilities and brucellosis is currently not routinely included as a priority disease within the IDSR system [16].

Techno-health as the application of information and communication technology (ICT)–based solution has been proposed to enhance early detection, timely reporting, and prompt response of brucellosis in humans using the AfyaData app [18]. It has additional features of supporting expert-authored materials such as guidelines and health tips that can be accessed by healthcare workers for immediate use as reference materials to enhance decision making process in clinical diagnosis and laboratory confirmation [18]. This study was carried out to evaluate the effect of awareness training on FHWs and the use of electronic based technology (AfyaData app) on reporting on human brucellosis cases in the selected pastoral communities.

## Materials and methods

### Study settings

**Fig. 1:**
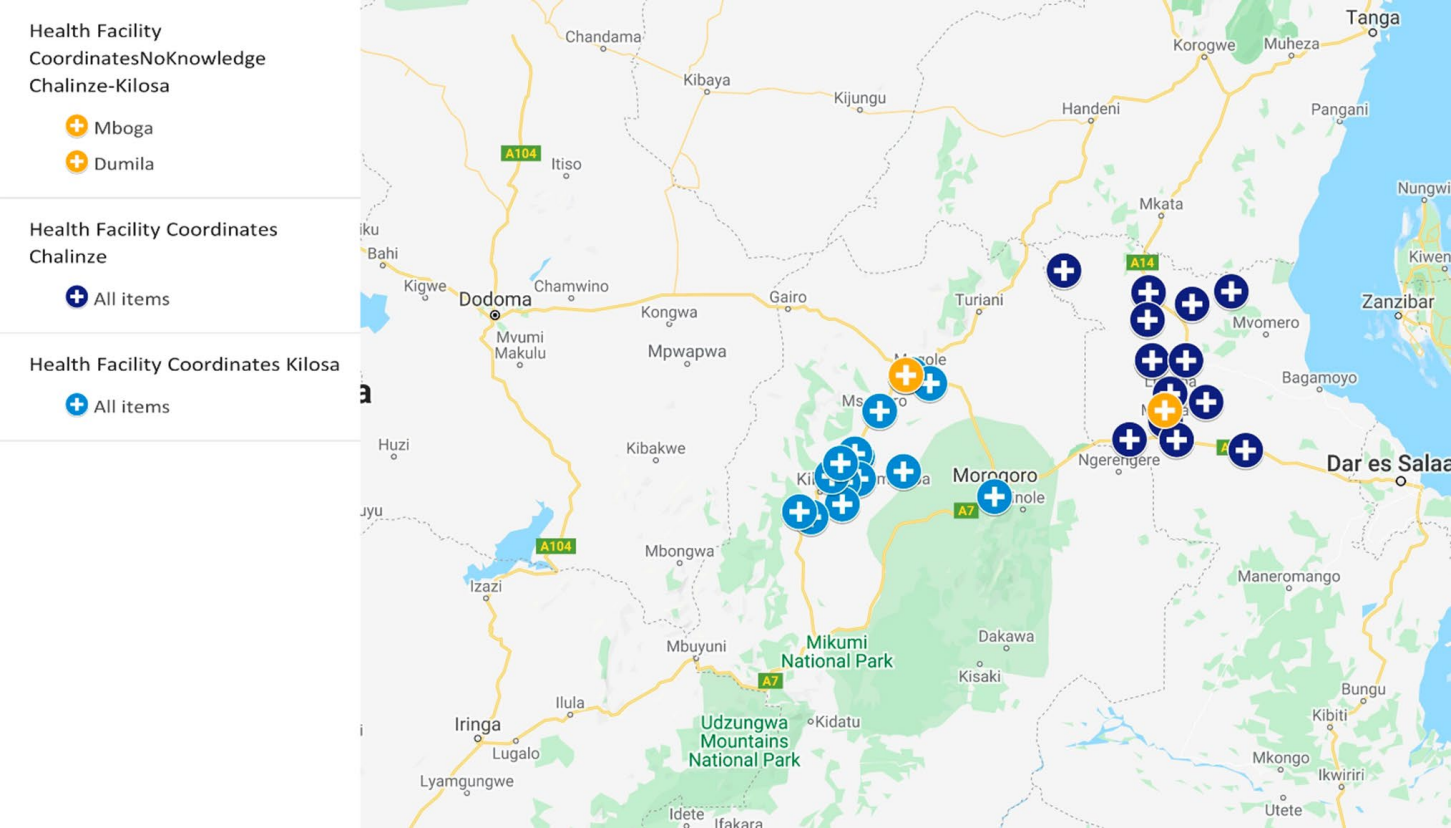
The map showing selected ward health facilities in control (Chalinze) and treatment (Kilosa) pastoral communities in Tanzania. Map created by ArcView GIS software version 3.2. Shapefiles for administrative boundaries from the 2012 census were sourced from the Tanzania National Bureau of Statistics

This study involved two pastoral communities (control and treatment) which were randomly selected in the eastern Tanzania. In both communities, agropastoralism is the main income activity [19]. The study areas were purposively selected as the areas with high population of pastoral communities keeping large population of domestic ruminants (cattle, sheep and goats). Administratively, Tanzania is divided into regions and each region is subdivided into districts. The districts are sub-divided into divisions and further into wards. The wards are further subdivided, for urban wards into streets and for rural wards into villages. Each ward is served with at least one primary health care facility (commonly a dispensary) and each village is served with at two Community Health Workers (CHWs) [14]. The figure below shows selected health facilities included in the study (Fig. 1).

### Study design and sampling techniques

The study adopted a quasi- experimental design which involved pretest-posttest design. The pretest was conducted in December 2019 [14], followed by a training which was conducted in May 2020 and a posttest in December 2020. The study involved two pastoral communities. In this study HWs and CHWs were referred as frontline health workers. The study involved random sampling of 16 wards from each pastoral community with large number livestock. In each ward one primary health facility, one HW and one CHW were purposively selected. Thus, 16 HWs and 16 CHWs from each pastoral community were selected with an overall of 64 frontline health workers (FHWs). The inclusion criteria to the study for the FHWs were, HWs who were medical in-charge of the facilities, with CHWs from the community being served by the selected health facility, owning a smartphone, working in a ward with large population of livestock and live within pastoral communities. All individuals seeking care at the outpatient’s department who were showing febrile symptoms in the selected health facilities in treatment pastoral community were eligible for brucellosis testing.

### Implementation steps

The study involved three implementation steps. The first step involved baseline data collection on knowledge, attitude, and practices of the FHWs regarding brucellosis management and reporting practices which served as control from December 2019 to January 2020 in both pastoral communities. The second step involved training of participants on the introduction about brucellosis, diagnosis using Rose Bengal test (RBT), how to handle the samples of suspected patients, using of *AfyaData* platform (mobile app) for reporting of brucellosis cases, receiving feedbacks and communicating results to patients from May 2020 to September 2020 in treatment community only. The last step involved collection of evaluation data of the training from the participants in both communities, conducted in December 2020.

### Intervention packages

#### Training on diagnosis and reporting

**Fig. 2:**
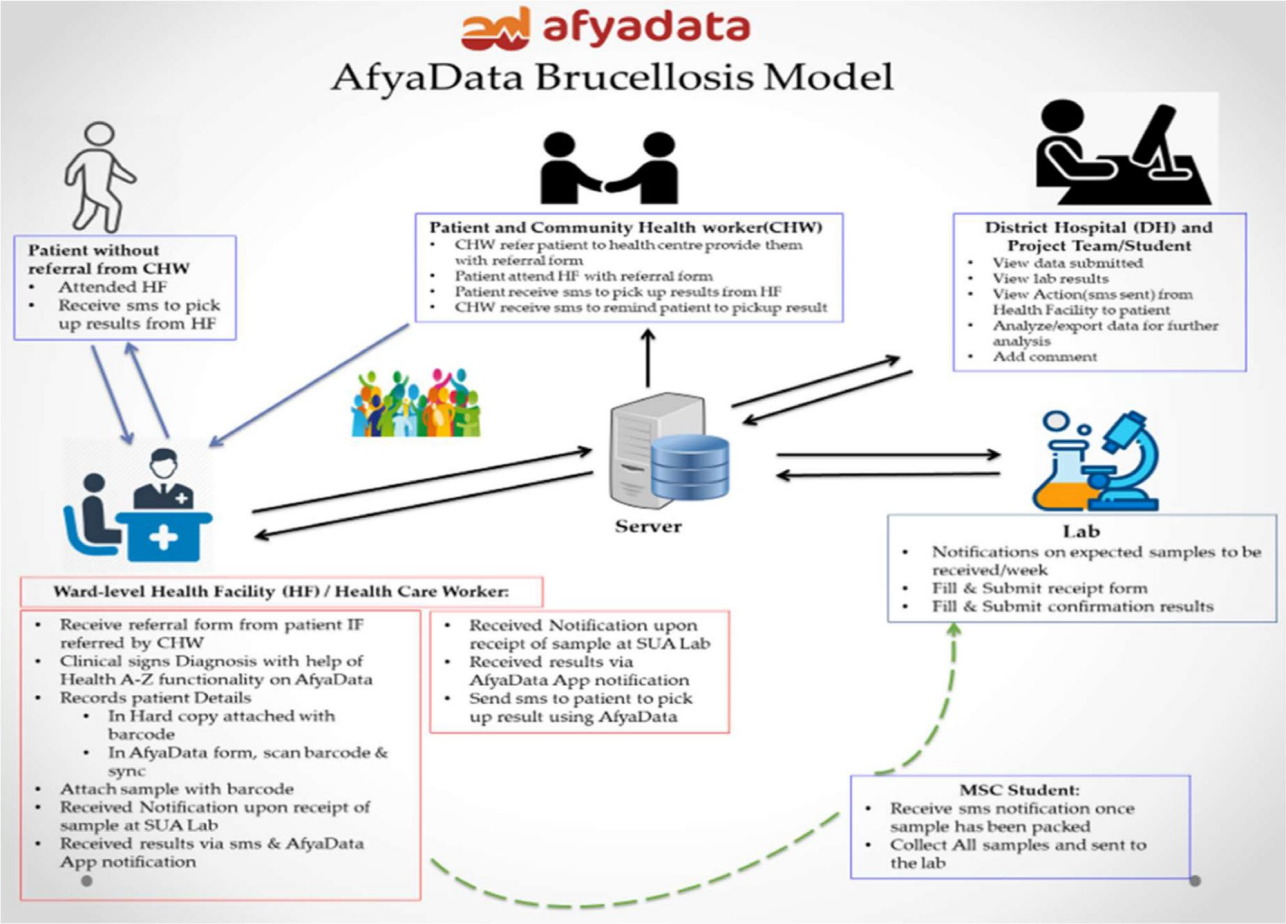
*AfyaData app* brucellosis model illustrating the reporting of samples from the health facilities up to the laboratory

The study adopted One Health approach which involved one epidemiologist, information technologist and public health officer as trainers. The training was prepared by the research team. It has used a plain teaching style of lectures and discussion. Participants were given training on the introduction, overview, transmission routes, symptoms, diagnosis, preventive measures, and treatment of brucellosis. The *AfyaData app* was set to support electronic-based training and awareness enrichment among FHWs to enhance early detection, timely presentation to health facilities and appropriate diagnosis of brucellosis cases. A laboratory component feature was developed on *AfyaData app* to track brucellosis samples from health facilities to main laboratory at the College of Veterinary Medicine and Biomedical Sciences, SUA, Morogoro, Tanzania (the laboratory facility is located approximately 140 km away from a treatment community), and shared results back to health facilities for near-to-real time access. In addition, the knowledge repository including the standard case definition for brucellosis was uploaded to *AfyaData app* platform and access was provided to health officials from the primary healthcare facilities to enhance the disease diagnosis. The functionality of the model to enhance tracking of samples and communication of laboratory test results was built using barcode feature embedded in the *AfyaData* platform. At the health facility, the patient meeting the standard case definition would be subjected to Rose Bengal test, which is a rapid diagnosis for brucellosis. A barcode with unique patient identification number was attached to the patient clinical assessment form that was presented to the laboratory at the health facility for Rose Bengal test. Once the RBT test was completed, the clinical management of the positive patients was initiated. Aliquots of RBT-positive serum samples in cryo-vials were refrigerated (2–8ºC) and transported in cool box with ice pack to a specialized laboratory at SUA where they were stored at -20ºC until confirmation of *Brucella* spp. exposure/infection using Enzyme-linked immunosorbent assay (ELISA). Using bar-coding system integrated in the *AfyaData* platform, laboratory results was sent back to respective health facility within 2–4 days so that proper management of suspected cases could start immediately using guidelines, which were developed by this study and distributed to the intervention site. Active interactions supported by *AfyaData app* was carried out between healthcare provider and patients either directly or through community-based volunteers in the respective areas, to enhance follow up of the patients’ course of medical condition and reminder messages on adherence to treatment regime (Fig. 2).

### Collection of samples and epidemiological data

#### Sample collection

Patients who attended the selected facilities with symptoms suggestive of brucellosis/ febrile illness were identified and their particulars were entered using the *AfyaData app*, barcodes were used as patient’s identification. A health personnel was assigned to aseptically collect 5 ml of blood from the patient’s brachial vein using a sterile disposable syringe into pre-labelled plain vacutainer tubes after relating their patient history with brucellosis clinical signs. The collected blood samples were centrifuged at 1500 g for 10 min to obtain sera. All collected sera were transferred to clean labelled cryovial. The cryovials were labeled using the same barcode as that from patient’s information to enable easy tracking of the samples. The tested sera were stored in a refrigerator until shipment to the college of Veterinary Medicine and Biomedical Sciences laboratory at Sokoine University of Agriculture where they were stored at -20°C^o^ until serological analysis.

### Demographic and KAP data collection

A structured questionnaire was uploaded in the *AfyaData app* in HWs smartphones in order to collect patient’s information on demographic data, variables on exposure to animals and animal products, consumption of raw milk and being in contact with aborted materials from animals. Also, a posttest structured questionnaire uploaded in the *AfyaData* platform was used to collect data after the training related to knowledge, attitude, and practices (KAP) of the HWs and CHWs. The information collected in posttest included socio demographic characteristics (age, gender, education, workstation experience and length of stay in position), knowledge of brucellosis (causes, mode of transmission, symptoms, diagnosis, treatment, and prevention), brucellosis practices (frequency of diagnosis, presence of reagents, types of samples for diagnosis, reporting practices and duration to receive feedback) and their attitude regarding brucellosis prevention and control. The questionnaire was prepared in English and translated to Swahili language, the national language so as to be understood by the majority.

### Laboratory analysis

#### Rose bengal test (RBT)

All collected sera samples were screened using Rose Bengal test (RBT) manufactured by APHA Scientific New Haw, Addlestone Surrey KT15 3NB, UK for *Brucella* antibodies according to the test procedure recommended by OIE [20] at the health facilities. Briefly, 20 µl of RBT antigen and 20 µl of serum sample using clean Pasteur pipette were placed onto the clean miscopy slide. Then the mixture was mixed by a sterile applicator stick. The mixture was then shaken manually for eight minutes before observation. The presence of pink granules (agglutination) was recorded as positive while a sample with no granules was recorded as negative. After RBT test both positive and negative samples were stored in the nearby health facility laboratory as recommended by OIE [20] for seven days, then transported in a cool box with ice packs to SUA and stored at -20ºС until c-ELISA was performed.

### Competitive enzyme linked immunosorbent assay (c-ELISA)

Both positive and negative sera were subjected to Competitive Enzyme-Linked Immunosorbent Assay (c-ELISA) a commercial kit SVANOVIR® BRUCELA-Ab c-ELISA as a confirmatory test, adopting a test procedure and interpretation of results as recommended by the manufacturer (Svanova Biotech AB SE-751 Uppsala, Sweden). Briefly, 45 µl of sample dilution buffer was placed in each well that was used for serum and control sample, respectively. A total of 5 µl of controls samples were added in duplicate in appropriate wells, followed by addition of 5 µl of dilution buffer into appropriate wells. Thereafter, 5 µl of test samples were added into each appropriate well. In addition, 50 µl of mAb-solution were added into all wells, followed by sealing the plate. Mixing of the reagents was done by placing the plate on the plate shaker. After incubation and rinsing four times with PBS-tween buffer, 100 µl conjugate solution were added to each well. After rinsing it again, 100 µl of substrate solution were added followed by incubation at room temperature for 10 min with subsequence addition of 50 µl of stop solution to each well. The optical densities (OD) of the controls and samples were measured at 450 nm in a microplate photometer (Micro read 1000, ELISA Plate Analyser) within 15 min after the addition of the stop solution to prevent fluctuation in OD values. The percent inhibition values (PI) for controls and samples were calculated using the formula defined by the ELISA kit manufacturer as here under:

PI = 100-(Mean OD _samples/Ctrl_ × 100).

Mean OD _conjugate control Cc_.

According to the c-ELISA kit manufacturer’s instructions, serum was regarded as positive if the PI value was > 30%. Only the patients that were tested positive in both RBT and c-ELISA were regarded as *Brucella* seropositive.

### Data analysis

The collected data from serological analysis and questionnaire survey were submitted to the server system located at the Sokoine University of Agriculture. For detailed analysis, data were exported from *Afyadata app* to the excel worksheet then to the Statistical Package for Social Science (SPSS) version 20.0. Descriptive analysis for frequencies, percentages, and proportion for both categorical and continuous variables were analyzed. The degree of association was analyzed using a Chi-square test/fisher test between brucellosis status with the sociodemographic information of the patients. FHWs knowledge and attitude regarding brucellosis after the training were assessed using five Likert scale, strongly agree to strongly disagree, and were assessed using scoring scheme, ranging from 5 to 1. The scores of the items were summed up and the total divided by number of the items giving a mean score for the part. The higher the mean score the better the knowledge and attitude. Knowledge was assessed using 36 Likert questions, one open question and one binary question (Yes/no). The overall score for Likert questions in knowledge was 180 i.e. (36*5). Attitude was assessed using nine Likert questions giving the overall score of 45 i.e. (9*5). In practice ten questions were measured by scoring method as follow yes option 1 score and no option zero score, *frequently* option 2 scores, *rarely* option 1 score and *none at all* option 0 score. The total score for practice was 16. Categorical variables were described by number and percent (N, %), where continuous variables described by mean and standard deviation (Mean, SD). Chi-square test and fisher exact test were used to compare between categorical variables while comparison between continuous variables was done by paired t-test to assess the effectiveness of the intervention. P-value less than 0.05 was considered significant.

### Ethical consideration

Ethical clearance was granted by the Medical Research Coordinating Committee of the National Institute of Medical Research in the United Republic of Tanzania (NIMR/HQ/R.8a/vol. IX/3235). Permission to conduct the study in the selected districts was obtained from the National, regional and local government authorities and Local Government in Morogoro (AB.175/245/01/219) and Pwani (DCD.128/40/01/109) regions. Written informed consent was obtained from each study participant. Confidentiality and identity protection were ensured throughout the analysis and interpretation of the study findings.

## Results

### Socio demographic information’s of the participants

A total of 64 frontline health workers were enrolled in the study. Their age ranged from 21 to 67 with an overall mean of 39.7 (± 1.382) years. Majority of participants were men 41 (64.1%) with the majority length of stay in their position ranged from 1 to 40 years. About 26 (40.6%) of the total participants had college education (Table 1).

**Table 1:** Participant’s sociodemographic characteristics

Variable	Total	Control, N = 32		Treatment, N = 32	
Sex	N = 64	Number	Percentage	Number	Percentage
Female	23	12	37.5	11	34.4
Male	41	20	62.5	21	65.6
**Age in years**					
21–30	19	6	18.8	13	40.6
31–40	18	13	40.6	5	15.6
41–50	15	7	21.9	8	25.0
> 50	12	6	18.8	6	18.8
**Education level**					
No formal education	3	2	6.3	1	3.1
Incomplete primary	1	1	3.1	0	0.0
Primary	23	12	37.5	11	34.4
Secondary	11	2	6.3	9	28.1
College	26	15	46.9	11	34.4
**Position**					
Medical doctor	2	2	6.3	0	0.0
Medical officer	3	2	6.3	1	3.1
Medical Assistant	17	9	28.1	8	25.0
Nurse	7	3	9.4	4	12.5
Laboratory technicians	3	0	0	3	9.4
Community health worker	32	16	50	16	50
**Length of stay in position (years)**					
1–10	34	20	62.5	14	43.8
11–20	13	7	21.9	6	18.8
21–30	11	3	9.4	8	25.0
31–40	6	2	6.3	4	12.5
**Working experience in workstation(years)**					
Less than a year	6	3	9.4	4	12.5
1–5 years	26	19	59.4	20	62.5
> 5 years	32	10	31.3	8	25.0

### Association between HWs sociodemographic with knowledge, practice and attitude mean scores about brucellosis in pastoral communities

There was a statistically significant difference between education, length of stay in position, years of experience in workstation and knowledge mean score in pre knowledge (p = 0.004,0.004, 0.002) respectively. On other hand, in pre practice statistically significant difference was observed in mean score between participants age level (p = 0.011) (Table 2).

**Table 2:** Association between HWs sociodemographic with knowledge, practice and attitude mean scores about brucellosis in pastoral communities

	Knowledge score		Practice score		Attitude score	
Variable	Pre knowledge	Post knowledge	Pre practice	Post-practice	Pre attitude	Post attitude
Gender	Mean ± SD	Mean ± SD	Mean ± SD	Mean ± SD	Mean ± SD	Mean ± SD
Female	0.73 ± 0.13	0.84 ± 0.1	0.1 ± 0	0.19 ± 0.15	0.95 ± 0.08	0.98 ± 0.05
Male	0.74 ± 0.24	0.91 ± 0.09	0.1 ± 0.06	0.23 ± 0.13	0.95 ± 0.11	0.97 ± 0.05
**P-value**	0.854	0.053	0.834	0.503	0.919	0.684
**Age in years**						
21–30	0.77 ± 0.09	0.89 ± 0.01	0.12 ± 0.04	0.3 ± 0.1	0.99 ± 0.04	0.95 ± 0.09
31–40	0.67 ± 0.26	0.89 ± 0.09	0.09 ± 0.03	0.2 ± 0.13	0.92 ± 0.1	0.98 ± 0.03
41–50	0.86 ± 0.05	0.89 ± 0.12	0.12 ± 0.04	0.2 ± 0.17	0.94 ± 0.09	1 ± 0
> 50	0.6 ± 0.52	0.9 ± 0.14	0.1 ± 0.06	0.2 ± 0.14	0.87 ± 0.23	1 ± 0
**P-value**	0.255	0.988	0.011*	0.446	0.175	0.254
**Education level**						
Secondary	0.52 ± 0.43	0.97 ± 0.05	0.1 ± 0.04	0.2 ± 0.14	0.97 ± 0.08	1 ± 0
College	0.79 ± 0.09	0.87 ± 0.1	0.1 ± 0.1	0.2 ± 0.21	0.94 ± 0.1	0.98 ± 0.05
**P-value**	0.004**	0.121	0.907	0.569	0.593	0.533
**Position**						
Medical doctor	0.8 ± 0	0.85 ± 0.07	0.1 ± 0	0.4 ± 0.07	0.95 ± 0.07	1 ± 0
Medical officer	0.67 ± 0.06	0.1 ± 0.06	0.1 ± 0	0.2 ± 0.12	0.9 ± 0.1	0.9 ± 0.1
Medical Assistant	0.74 ± 0.21	0.88 ± 0.09	0.1 ± 0.02	0.2 ± 0.12	0.94 ± 0.09	0.98 ± 0.05
Nurse	0.71 ± 0.32	0.87 ± 0.15	0.1 ± 0.08	0.2 ± 0.19	0.94 ± 0.15	1 ± 0
Laboratory technicians	0.83 ± 0.12	0.87 ± 0.06	0.2 ± 0.06	0.3 ± 0.17	1 ± 0	1 ± 0
**P-value**	0.900	0.660	0.194	0.408	0.835	0.065
**Length of stay in position(years)**						
0–10	0.78 ± 0.09	0.89 ± 0.09	0.1 ± 0.03	0.2 ± 0.12	0.97 ± 0.07	0.98 ± 0.06
11–20	0.5 ± 0.41	0.88 ± 0.08	0.06 ± 0.05	0.1 ± 0.14	0.92 ± 0.1	0.98 ± 0.04
21–30	0.9 ± 0	0.77 ± 0.15	0.1 ± 0.1	0.3 ± 0.2	0.87 ± 0.23	1 ± 0
**P-value**	0.004**	0.107	0.099	0.081	0.189	0.809
**Working experience in workstation(years)**						
Less than a year	0.4 ± 0.46	0.88 ± 0.13	0.05 ± 0.06	0.25 ± 0.17	1 ± 0	0.9 ± 0.12
1–5 years	0.79 ± 0.11	0.89 ± 0.11	0.11 ± 0.04	0.21 ± 0.15	0.93 ± 0.1	0.99 ± 0.03
> 5 years	0.8 ± 0	0.86 ± 0.07	0.1 ± 0	0.2 ± 0.12	1 ± 0	1 ± 0
**P-value**	0.002**	0.634	0.048*	0.849	0.352	0.002**

* ANOVA test significant difference at p. value < 5%, **< 1%, % pre = pre-training, Post = post-training

### Association between CHWs sociodemographic with knowledge, practice and attitude mean scores in pre and post intervention regarding brucellosis in pastoral communities

There was highly statistically significant difference in knowledge in CHWs with less than a year compared to those with more than one-year experience in working station (p = 0.006) in post education. A significant difference in attitude was observed in CHWs with age group 21–30 and 31–40 years than other age group (p = 0.031). Also, it showed that there was statistically difference in reported practices between education level and pre practice mean score (p = 0.031) (Table 3).

**Table 3:** Association between CHWs sociodemographic with knowledge, practice and attitude mean scores in pre and post intervention regarding brucellosis in pastoral communities

		Knowledge score		Practice score		Attitude score
Variable	Pre-knowledge	Post knowledge	Pre-practice	Post-practice	Pre-attitude	Post attitude
Sex	Mean ± SD	Mean ± SD	Mean ± SD	Mean ± SD	Mean ± SD	Mean ± SD
Female	0.39 ± 0.35	0.71 ± 0.18	0.09 ± 0.24	0.3 ± 0.39	0.51 ± 0.03	0.75 ± 0.18
Male	0.21 ± 0.31	0.7 ± 0.15	0.01 ± 0.04	0.15 ± 0.23	0.53 ± 0.15	0.73 ± 0.18
**P-value**	0.209	0.88	0.135	0.174	0.679	0.859
**Age in years**						
21–30	0.65 ± 0.07	0.74 ± 0.21	0 ± 0	0.2 ± 0.28	0.3 ± 0.42	0.8 ± 0.28
31–40	0.36 ± 0.34	0.74 ± 0.2	0.02 ± 0.06	0.13 ± 0.15	0.54 ± 0.1	0.78 ± 0.19
41–50	0.16 ± 0.28	0.7 ± 0.15	0.01 ± 0.03	0.25 ± 0.29	0.56 ± 0.09	0.75 ± 0.18
> 50	0.21 ± 0.34	0.66 ± 0.13	0.09 ± 0.25	0.21 ± 0.42	0.5 ± 0	0.68 ± 0.17
**P-value**	0.185	0.691	0.602	0.881	0.031*	0.647
**Education level**						
No formal education	0.26 ± 0.46	0.7 ± 0.17	0.27 ± 0.46	0.33 ± 0.49	0.5 ± 0	0.73 ± 0.23
Incomplete primary	0.7±	0.9±	0 ± 0	0.4±	0.6±	1±
Primary	0.24 ± 0.31	0.69 ± 0.15	0.02 ± 0.05	0.2 ± 0.3	0.51 ± 0.14	0.71 ± 0.17
Secondary	0.28 ± 0.38	0.74 ± 0.19	0 ± 0	0.08 ± 0.17	0.58 ± 0.08	0.8 ± 0.2
**P-value**	0.607	0.577	0.031*	0.624	0.624	0.378
**Length of stay in**						
**position (years)**						
0–10	0.4 ± 0.33	0.72 ± 0.16	0.02 ± 0.06	0.19 ± 0.23	0.49 ± 0.18	0.8 ± 0.19
11–20	0.1 ± 0.26	0.7 ± 0.17	0.01 ± 0.04	0.2 ± 0.4	0.5 ± 0	0.67 ± 0.15
21–30	0.21 ± 0.29	0.67 ± 0.15	0 ± 0	0.16 ± 0.29	0.59 ± 0.13	0.71 ± 0.16
31–40	0.25 ± 0.39	0.72 ± 0.18	0.15 ± 0.32	0.27 ± 0.36	0.52 ± 0.04	0.73 ± 0.21
**P-value**	0.245	0.946	0.215	0.939	0.381	0.504
**Working experience**						
**in workstation(years)**						
Less than a year	0.4 ± 0.35	0.97 ± 0.06	0 ± 0	0.3 ± 0.26	0.5 ± 0	0.9 ± 0.17
1–5 years	0.25 ± 0.33	0.68 ± 0.16	0.02 ± 0.06	0.19 ± 0.33	0.55 ± 0.08	0.74 ± 0.19
> 5 years	0.25 ± 0.34	0.67 ± 0.13	0.06 ± 0.2	0.19 ± 0.29	0.51 ± 0.16	0.71 ± 0.17
**P-value**	0.757	0.006**	0.63	0.839	0.668	0.234

* ANOVA test significant difference at p. value < 0.05, **<1%

### Correlation between pre- and post-intervention knowledge, attitude, and practice for the participants

A paired-samples t-test was conducted to compare the mean score of knowledge, attitude, and practice of the treatment group and control group of the HWs and CHWs, before the intervention and after the intervention. There was a significant correlation in HW practice (p = 0.016) and attitude (p = 0.006) mean scores, in CHWs knowledge (p = 0.003), practice (p = 0.004) and attitude (p = 0.000) mean scores. Also, a significant increase was found in knowledge scores of HWs (p = 0.003), practice score (p < 0.001) and attitude score (p = 0.032). Also, in CHWs mean score of knowledge (p < 0.001), practice (p = 0.001) and attitude (p < 0.001) after the intervention.

### Brucellosis seropositivity in febrile patients in treatment pastoral community

A total of 141 serum samples from patients were screened by RBT, where by 17 (12.1%) were positive and confirmed by c-ELISA, and four (2.8%) were positive in the selected ward health facilities in treatment pastoral community.

### Brucellosis cases reported through electronic tool (*AfyaData app*)

A total of 141 brucellosis cases, based on clinical diagnosis were reported in four consecutive months in treatment pastoral community using *AfyaData app* from May to September 2020, in patients with febrile illnesses seeking health care. Only six (4.3%) cases were reported by the CHWs using referred forms. Most of the cases were from Kimamba health facility 28 (19.9%). More than half, 93 (66.0%) of disease cases reported were from females. Half of the patients who were seropositive were from Msowero health facility. Significant association was found between number of cases in health facilities and seropositivity (p = 0.0003) (Table 4).

**Table 4:** Brucellosis cases reported through *AfyaData* app in treatment community from May to September 2020 with their socio demographic information

Variable	N = 141	Frequency (n%)	Seropositive (RBT),	P-value	Seropositive (c-ELISA), n (%)	P-value
			n (%)			
**Sex**	Male	48 (34.0)	7 (41.2)	0.588	1 (25.0)	1.000
	Female	93 (66.0)	10 (58.8)		3 (75.0)	
**Age**	1–10	15 (10.6)	0 (0.0)		0 (0.0)	
	11–20	23 (16.3)	4 (23.5)	0.543	1 (25.0)	0.711
	21–30	32 (22.7)	5 (29.4)		0 (0.0)	
	31–40	34 (24.1)	4 (23.5)		2 (50.0)	
	> 40	37 (26.2)	4 (23.5)		1 (25.0)	
**Number of cases in health facility**						
	Kimamba	28 (19.9)	2 (11.8)		0 (0.0)	
	Rudewa	6 (4.3)	5 (29.4)	**0.0003**	0 (0.0)	0.336
	Msowero	19 (13.5)	1 (5.9)		2 (50.0)	
	Tindiga	2 (1.4)	0 (0.0)		0 (0.0)	
	Dakawa	9 (6.4)	0 (0.0)		0 (0.0)	
	Dumila Dispensary	26 (18.4)	1 (5.9)		0 (0.0)	
	Dumila HC	7 (5.0)	1 (5.9)		1 (25.0)	
	Dodoma Isanga	1 (0.7)	0 (0.0)		0 (0.0)	
	Changarawe	1 (0.7)	0 (0.0)		0 (0.0)	
	Twatwatwa	4 (2.8)	0 (0.0)		0 (0.0)	
	Serengeti kada	2 (1.4)	0 (0.0)		0 (0.0)	
	Agape	11 (7.8)	5 (29.4)		0 (0.0)	
	Chanzuru	10 (7.1)	0 (0.0)		0 (0.0)	
	China Estate	15 (10.6)	2 (11.8)		1 (25.0)	

Statistically significant (p < 0.05) P-value is highlighted in bold

### Observed clinical signs in brucellosis seropositive febrile patients in treatment pastoral community

Several clinical manifestations were recorded from febrile patients attending selected health facilities are presented in the Table 5. Headache was the most self-reported clinical manifestation by respondents (20.8%) followed by fever (13.3%), joint pain (13.3%), and fatigue (9.9%).

**Table 5:** Observed clinical signs in brucellosis seropositive febrile patients in treatment pastoral community

Clinical signs (n = 141)	Frequency	(%)
Headache	80	20.8
Fever	51	13.3
Joint pain	38	9.9
Fatigue	36	9.4
Vomiting	26	6.8
Body weakness	25	6.5
Loss of appetite	24	6.20
Coughing	22	5.7
Sweating at night	22	5.7
Diarrhea	12	3.1
Nausea	11	2.9
Back pain	9	2.3
Abortion	8	2.1
Abdominal pain	7	1.8
Bitter mouth	5	1.3
Weight loss	4	1.0
Muscle pain	4	1.0

## Discussion

The current study assessed the effectiveness of the training on awareness and the use of electronic tool on reporting of brucellosis in frontline health workers in pastoral communities. The findings revealed that majority of frontline health workers were not aware of brucellosis before the intervention. Meanwhile, four months after the intervention their awareness was improved. Regarding FHWs socio characterictics, more than three quarter of the HWs had college education while majority of the CHWs had primary education which is generally low level of education. The low education level of CHWs might had influenced awareness and practices towards control of brucellosis in these pastoral communities. Similar results were found in KAP study for animal health and public health workers conducted in Sudan and northern Uganda [21, 22]. The study also, revealed that duration at work, education and experience of the HWs had a positive impact on their knowledge of brucellosis. This might be due to exposure and training workshops, or seminars participated during their profession work. Although the mean score of knowledge differed with position of the HWs but the difference was not significant. The nurses mean score was low compared to the medical doctors. In Tanzanian situation, most of the health facilities serving the communities are managed by nurses. It could be possible that some patients who attend the facilities may be suffering from brucellosis but due to their low awareness on the disease proper diagnosis and treatment of the disease can be hindered [22].

On the other hand, the study revealed low awareness in CHWs regarding brucellosis in both communities before the training. The poor knowledge among CHWs could be attributed to their low level of education and inadequate public health promotion regarding zoonotic diseases. This observation might have affected the detection and management of brucellosis in communities they serve. Similarly, low knowledge about the disease and risky practices among the community health workers have been reported in China and South Africa [23, 24]. It was observed that the mean score of knowledge in pretest and post intervention was higher in CHWs with age group of 21–30 and 31–40 years than other age group and for women CHWs the knowledge mean score was higher than males, but the difference were not significant.

The findings of the study revealed that the reporting practices of brucellosis before the intervention were very poor in both communities. Meanwhile, majority of participants in treatment pastoral community were found to have better practices than control community [25].

The study also revealed a highly significant correlation between HWs mean scores in pretest and posttest in practice and attitude. Also, a highly significant increased between mean score of pretest and posttest in knowledge, practice and attitude. This indicates that the intervention conducted was effective in improving the knowledge, practices, and attitudes of the FHWs. Majority of HWs practices improved after intervention, they were found to consider brucellosis during diagnosis, also report the disease cases using the electronic technology, receive the feedback and communicate them to the patients. This has enabled the quick reporting of the disease cases in the treatment pastoral community and raised awareness of disease diagnosis while improving their attitude regarding brucellosis prevention and control measures. Increased knowledge, practices and attitudes were similarly observed in studies conducted in Egypt in nurses and slaughterhouse workers when assessed the effectiveness of health educational program [25, 26].

Also, mean score of knowledge and attitude regarding brucellosis for CHWs was found to increase significantly after the training. The CHWs were found more knowledgeable on transmission, symptoms, and preventive measures of brucellosis. The mean score of practices was reported to increase significantly after the intervention, this implies that CHWs from treatment pastoral communities were advising the farmers to screen their animals for brucellosis and, they were found to provide referral form to patients with symptoms suggestive of brucellosis. The results could provide guidance on formulation of strategies to improve early detection and management of brucellosis in the study districts and other similar settings.

The results of the study indicate that the mobile phone reporting application has revitalized the reporting of brucellosis cases in selected wards in treatment pastoral community. By using this electronic based system (*AfyaData app*) [18] for reporting brucellosis cases, a total of 141 brucellosis cases in human were reported in the first four months in treatment pastoral community. This may be due to the fact that the application was easy to navigate and quick to learn for the HWs. The training was not time consuming and the fact that recording of reports from patients does not require internet connection it makes the process easier. In all the cases reported few patients were referred by CHWs through referral forms. This may due to the majority of CHWs didn’t receive the training.

Application of mobile phones and ICT technologies to improve disease reporting and surveillance in public health has been reported in Tanzania for other zoonotic diseases like rabies [27]. Also, it has been used in other countries such as, China [28], Sri Lanka [29], Zambia, Uganda and Madagascar [30], and Kenya [31]. Combining the participatory community based approaches with mobile technology has the potential to support not only early detection of disease events that are happening at the community level but also actual response [32].

In this study no association was demonstrated in gender and age of the patients with brucellosis seropositivity, this may be due to small number of febrile patients recruited and majority of them being female of similar age. These findings are similar to other studies that reported lack of association between sex and age with brucellosis positivity [33]. Significant association was found in relation to location (ward health facilities). This clearly indicates that the number of patients with seropositivity in *Brucella* infection was concentrated in certain wards. The high exposure rate was found in Rudewa ward, this may be attributed by the fact that the ward is inhabited with high number of Maasai community whose livelihood depend much on livestock keeping increasing the risk of acquiring brucellosis.

During screening of febrile patients in the selected health facilities it was found that higher seropositive rate/clinical rate was from female patients, but the difference was not statistically significant. This can be explained by the fact that in pastoral and most agro-pastoral setup, females do most of the work associated with livestock such as milking, cleaning of livestock houses, and handling of the newly borne calves, which may predispose them to the infection. Furthermore, in African settings, most women attend health facilities and hence possibilities of being diagnosed with different diseases which may be the case for them having high number of diagnosed brucellosis cases [34]. Similar findings were found in Ngorongoro district in northern Tanzania by [35] and also, in Kampala, Uganda by [36]. These findings are contrary with the study conducted in Mbeya by [37] who reported zero (0%) seropositivity in females and higher in males, also in Rwanda [33] reported higher rate in males compared to females. Differences in prevalence rates between the sexes may be attributed to different behavioral attitudes towards livestock handling and preparation of food of animal origin in those communities [35].

In this study it was found that most of seropositive patients were in age group of 31–40 and above 40 years, these are considered as adults who were associated with livestock keeping hence longer exposure time and higher risk of acquiring brucellosis. Similar results were reported by [36, 38]. Another group which was found affected ranged from 11 to 20 years; these are teenagers who in the pastoralist communities are more associated with herding animals, milking and also help during delivery when animals are in the pastoral area. Most of them lack knowledge in handling animals and aborted materials when assisting animals in delivery so this puts them in higher risk of contracting brucellosis. Similar results were found in the study conducted in febrile patients in Kenya and northern Tanzania by [39].

### Conclusion and recommendation

Based on the findings of the present study it can be concluded that before the intervention the participants knowledge and practices on brucellosis were low, although they showed good attitude. Meanwhile, after the training intervention participant’s knowledge, attitude and practices improved significantly. Brucellosis was diagnosed in some febrile patients in treatment pastoral community but in low prevalence. There is a need for the use of electronic-based technology to improve timely management of brucellosis in pastoral communities. Also, continuous training on FHWs regarding the disease is needed to improved their awareness and practices.

### Electronic supplementary material

Below is the link to the electronic supplementary material.

**Supporting information**: Appendix 1: Ethical consideration. Appendix 2: Questionnaire used for the study

## Data Availability

The datasets used and/or analyzed during the current study are available from the corresponding author on reasonable request.

## List of abbreviations

SPSS: Statistical package for the social sciences
HWs: Healthcare workers
FHW: Frontline health workers
CHW: Community health workers
KAP: Knowledge, Attitude and Practices
SD: Standard deviation
SAT: Standard tube Agglutination Test
OIE: World Organization for Animal Health
WHO: World Health Organization
